# Insights from Team Clinic: A Person-Centered Virtual Peer Group Care Model Adapted for Marginalized and Historically Excluded Youth with Type 1 Diabetes (T1D)

**DOI:** 10.3390/children11111383

**Published:** 2024-11-14

**Authors:** Jaquelin Flores Garcia, Mark W. Reid, Alejandra Torres Sanchez, Valerie Ruelas, Sarah-Jeanne Salvy, Alex Thomas, Gary Ashwal, D. Steven Fox, Jennifer K. Raymond

**Affiliations:** 1 Children’s Hospital Los Angeles, Los Angeles, CA 90027, USA; mreid@chla.usc.edu (M.W.R.); atorressanchez@chla.usc.edu (A.T.S.); vruelas@usc.edu (V.R.); jraymond@chla.usc.edu (J.K.R.); 2 Division of Endocrinology, Keck School of Medicine of USC, Los Angeles, CA 90033, USA; 3 Research Center for Health Equity, Cedars-Sinai Medical Center, Los Angeles, CA 90048, USA; sarah.salvy@cshs.org; 4 Booster Shot Media, Venice, CA 90291, USA; alex@boostershotmedia.com (A.T.); gary@boostershotmedia.com (G.A.); 5 Department of Pharmaceutical & Health Economics, USC Alfred E. Mann School of Pharmacy and Pharmaceutical Sciences, Los Angeles, CA 90089, USA; steven.fox@med.usc.edu

**Keywords:** youth, type 1 diabetes, marginalized and historically excluded, virtual peer groups, person-centered care

## Abstract

Background: Despite advancements in T1D care regimens, racially and ethnically diverse youth with low income continue to experience worse health outcomes, more psychosocial challenges, and higher barriers to care. Alternative care models are needed to address the needs of this population. Methods: Team Clinic is a person-centered virtual peer group (VPG) care model that was assessed in a 15-month, pragmatic randomized controlled trial. Youth (ages 10–17) and their families were assigned to study arms based on their clinician’s group (standard care or person-centered care, PCC) and then randomized to VPGs or no groups. Results: Data from 79 youth and their families were examined. While positive outcomes were seen across all study groups, youth that participated in Team Clinic (PCC + VPG) reported the largest increases in resilience at the end of the study (+7.42, to 51.63, *p* = 0.009). These participants also reported the lowest levels of depressive symptoms as assessed by PHQ-8 scores (−5.07, *p* = 0.002) at the end of the study. Conclusion: Team Clinic can serve as an alternative care model for racially and ethnically diverse youth with T1D and their families. VPGs can provide unique benefits, including an increase in resilience, a decrease in depressive symptoms, and a safe space for families to connect, learn, and receive support.

## 1. Introduction

In 2021, approximately 304,000 adolescents (ages 10–19 years) were diagnosed with type 1 diabetes (T1D) in the United States (US) [1]. Over the previous two decades, the incidence of T1D has been increasing in adolescents from racially and ethnically diverse, marginalized, and/or historically excluded populations, and this trend continues today [2]. Despite advancements in available insulin (e.g., faster- and longer-acting) and diabetes technology (e.g., continuous glucose monitors (CGMs), insulin pumps, automated delivery systems), ethnically and racially diverse adolescents with low income often do not have access to these innovations, impeding their ability to obtain or maintain optimal glycemic levels [3,4]. Specifically, Latinx and Black adolescents with T1D use CGMs and insulin pumps less frequently than their non-Latinx white peers and experience more elevated hemoglobin A1c (HbA1c) [4]. Across all ages, racially and ethnically diverse groups with T1D who use public insurance are more likely to have elevated HbA1c than those who use private insurance [4]. In addition, adolescents from diverse groups with lower socioeconomic status (SES) are more likely to be impacted by psychosocial challenges to managing T1D, like higher levels of diabetes distress [5,6], food insecurity, unstable housing, and other social determinants of diabetes health [7,8,9,10,11]. Within the health care system, negative experiences with discrimination, racism, and clinician bias remain prevalent deterrents to diverse groups accessing the intensive regimens and diabetes technology integral to standard care [12,13,14].

Racially and ethnically diverse adolescents still meet the ADA recommendation for HbA1c (<7%) less frequently, perform self-monitoring blood glucose (SMBG) checks less often than recommended (6–10 times), or do not attend quarterly diabetes care appointments as routinely as their non-Latinx white peers [15,16]. Diverse groups remain understudied [6], but it is clear that there is a need for alternative care models that provide support in addition to what is offered by standard care. To date, few innovative T1D clinical care models have been specifically designed and adapted for racially and ethnically diverse, marginalized, and historically excluded populations.

Designed to improve patient access while addressing increasing demands on clinicians’ time, shared medical appointments (SMAs) have been found to strengthen patient and clinician satisfaction [17], increase clinic efficiency [18,19], and improve outcomes and self-management in adults living with diabetes and other conditions. SMAs have been successfully expanded to youth living with diabetes and their families, with studies showing that youth learn more and discuss more diabetes-related topics [20], improve their quality of life, and gain greater responsibility for care [21] when attending SMAs. SMAs have also been adapted for Latinx populations living with diabetes, with research showing increased uptake of diabetes technology, high satisfaction, and some improvements in glycemic control [22].

In 2013, seeing a critical need for innovative diabetes care models, a multidisciplinary team of social workers, dietitians, diabetes nurse educators, and endocrinologists designed Team Clinic, a person-centered, peer-group-based care model for T1D [23]. Team Clinic differs from standard care by using the SMA model, where youth and their families participate in diabetes support and education groups led by one or more healthcare professionals, compared to one-on-one visits between a single clinician and youth in standard care. Further, Team Clinic clinicians are trained in motivational interviewing and the utilization of shared decision-making tools to enhance person-centered care [24]. The Team Clinic model was initially pilot-tested at a large pediatric diabetes center in Aurora, Colorado [23]. In this pilot, participants were predominantly non-Latinx white families (youth ages 13–18 and family members) with private insurance. After participating in Team Clinic, youth and their family members reported feeling more supported and understood than in their routine care visits [23,25]. Youth in Team Clinic also reported reduced family conflict and improved psychosocial outcomes (e.g., better mood, sleep, appetite) in comparison to the control group [26]. Clinicians indicated that Team Clinic allowed them to provide much-needed education to families, and most clinicians thought Team Clinic enabled them to provide higher quality care [23,25].

Due to its success, the Team Clinic model was adapted for Children’s Hospital Los Angeles (CHLA), an urban outpatient diabetes care center primarily serving racially and ethnically diverse families [24]. Over 70% of the patients and families served at CHLA receive public insurance, and over 80% are part of communities of color. During Phase 1 of the project, the research team conducted focus groups, including patients, family members, and teachers; their feedback was used to design the Team Clinic toolkit and the peer groups [24]. The toolkit curriculum was designed to help adolescents gain skills and confidence as they transition to a more active role in managing their diabetes, while also providing diabetes care teams the resources needed to successfully teach these skills. Team Clinic was originally designed to be delivered in person; however, due to the COVID-19 pandemic, the intervention was adapted to be delivered either virtually or in person (e.g., in-person peer groups became virtual peer groups (VPGs)). This manuscript reviews the results of Phase 2, a pragmatic randomized controlled trial (RCT) of the manualized virtual and in-person intervention, including clinical outcomes and participant satisfaction. This trial was registered on ClinicialTrials.gov (NCT04190368).

## 2. Materials and Methods

### 2.1. Study Design and Participant Criteria

**Figure 1:**
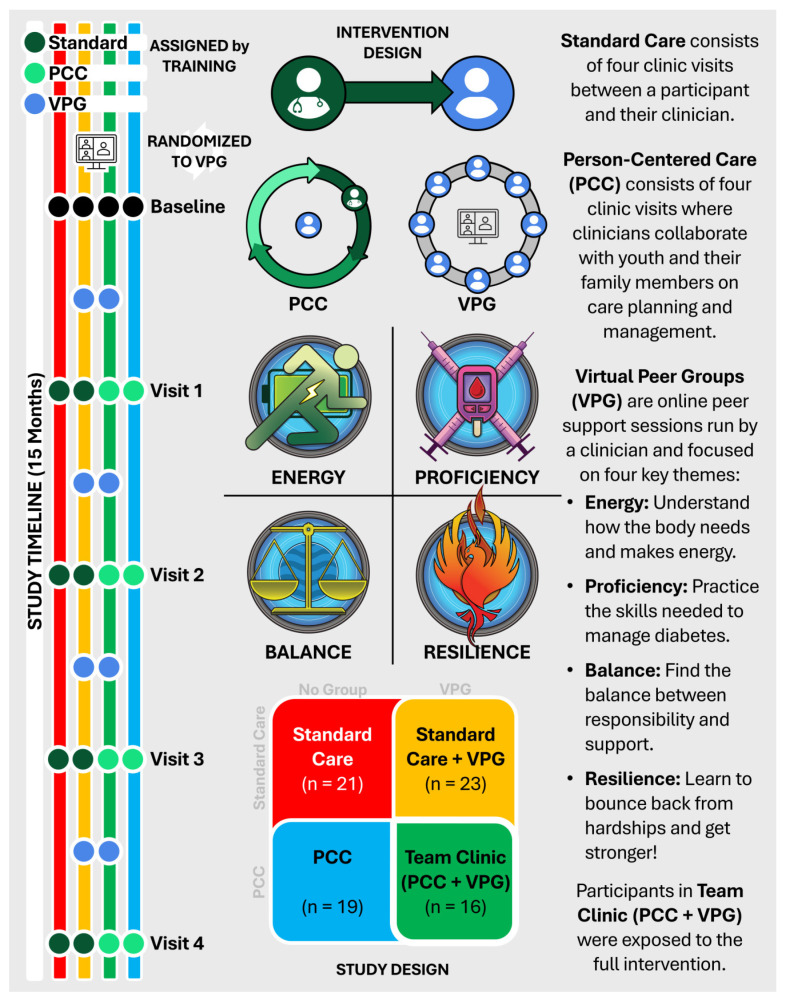
Team Clinic intervention and study design.

This study was a 15-month, pragmatic RCT including youth, their families, and their clinicians. Youth and their families were assigned to study groups based on their clinician’s orientation and training (standard care or person-centered care) and then randomized into virtual peer groups or no groups (see 2 × 2 study design, Figure 1). The full Team Clinic intervention includes person-centered care and VPGs. English-speaking youths were eligible if they were between the ages of 10 and 17 with T1D for at least 6 months, in 6th–12th grades, not currently in other group interventions, receiving or pending care at CHLA, and had California Children’s Services (CCS)/self-pay and/or private insurance. Their families were eligible if they were their primary caregiver, ≥18 years old, and English-speaking. The youth and their family were not eligible if they did not speak English, had severe psychosocial diagnoses, behavioral or developmental disabilities, or any comorbidities (e.g., cystic fibrosis, uncontrolled thyroid disease) that would make group participation difficult. At the study’s end, all youth and family members were invited to provide feedback via focus groups about the intervention.

### 2.2. Clinician Training (Standard Care or Person-Centered Care)

Clinicians in standard care provided care as usual. Team Clinic clinicians were trained to deliver person-centered care (PCC), focusing on how care delivery impacts the health and wellness of youth with T1D. One-hour clinician trainings occurred biweekly via the WebEx teleconferencing platform [27] over four months, followed by self-guided content review by clinicians as needed. Topics included “Spirit of Motivational Interview,” “Open-ended questions, Affirmations, Reflections, and Summaries,” “Eliciting Change Talk,” and “Challenging Situations.” Team Clinic clinicians participated in role-playing exercises (e.g., reflection volleys) and were trained on how to fill out shared decision-making tools for appointments, which were used to set care priorities and diabetes appointment agendas with youth and their family members. Following informed consent, the clinical research coordinators (CRCs) introduced these materials to the youth and their family members. Care plans were developed together by clinicians, youth, and their families, and they included specifying the responsibility of all participants. At appointments, youth and families had the option to review supplies and medications, blood sugar ranges, feelings and thoughts about care, social or family concerns, care complications, and other issues as needed. Family members also discussed accomplishments and challenges related to their youth’s T1D.

### 2.3. Virtual Peer Groups

Virtual peer groups were peer support groups held virtually every 3–5 months via WebEx. Each one-hour session was facilitated by a clinical team member (social worker or registered dietician) and focused on one of four themes: energy (physical activity), proficiency (diabetes management skills), resilience (managing obstacles and challenges), and balance (managing responsibilities and being mindful) (see Figure 1). Youth sessions included diabetes education and discussion of the psychosocial impacts of T1D and care. Family member sessions featured the same themes but focused on how well youth and family were handling peer discussions, providing tips for managing emotions and other psychosocial support. After eight participants were randomized, VPG sessions were scheduled based on participant availability. Participants were allowed to attend any session they missed as more participants were randomized and additional sessions were made available. CRCs reminded participants of sessions via email and text, offered technical support to connect youth and family members to virtual groups, and were present during sessions to assist the facilitator and/or troubleshoot any technical issues. Youth and their family participated in separate groups that were held simultaneously or staggered. Participants were encouraged to have diabetes kits (e.g., glucose tablets, glucagon, juice) nearby, since some sessions involved physical activities.

### 2.4. Recruitment for Study

CRCs conducted weekly and monthly electronic health record (EHR) queries that included all youth with T1D scheduled for clinic appointments. The CRCs would thoroughly review each potentially eligible youth prior to approaching them and their family in the clinic or remotely (phone, text, and/or email). Clinicians were also able to refer their patients, and the CRCs would follow the same screening procedures. Acknowledging the unique needs of our diverse families, the team set persistent recruitment goals, and each family was contacted up to eight times [28]. CRCs reviewed study information with both the youth and their family, and if they were interested in participation, the CRC would either obtain their consent in the clinic or schedule a remote consent conference. Families had the opportunity to ask questions about the study during all stages of the consent process.

### 2.5. Youth, Families, and Clinician Outcomes and EHR Data

After randomization, youth and their family members completed surveys at the baseline and after 3, 6, 9, and 12 months via REDCap [29]. A hemoglobin A1c (HbA1c) kit was provided to the youth if an HbA1c value was not obtained as part of standard care within the last 30 days of the screening visit. EHR data were abstracted at each of these timepoints. Clinicians completed the baseline and all visit surveys via REDCap [29].

### 2.6. Youth and Family Reported Outcomes

Self-reported and EHR data were collected at the baseline and on a quarterly basis. Participants completed six validated measures:

The Patient Health Questionnaire-8 (PHQ-8) is a widely used, standardized, and validated self-report questionnaire to assess depressive symptoms and their severity [30]. The PHQ-8 captures eight symptoms similar to the DSM-IV diagnostic criteria for Major Depression Disorder (MDD)—little interest or pleasure, depressed mood, sleep disturbances, tiredness or little energy, poor appetite or overeating, feelings of worthlessness or guilt, trouble concentrating, and psychomotor retardation or agitation. Items are rated on a 0–3 scale (0 = not at all to 3 = nearly every day) and totaled. A score of 0 to 4 indicates no depressive symptoms, 5 to 9 indicates mild depressive symptoms, 10 to 14 is moderate, 15 to 19 is moderately severe, and 20 to 24 is severe [30]. Scores of 10 or greater are consistent with a diagnosis of MDD, and scores of 15 or greater suggest Severe MDD, although no formal diagnoses were made during this study.

Problem Areas in Diabetes (PAID-T) is a 26-item self-report questionnaire to assess diabetes-specific distress (emotional, food-related, social, and treatment-related) in teens and their caregivers [31]. Items are rated on a 6-point Likert scale (1 = not a problem to 6 = serious problem) and totaled. Higher scores indicate greater distress. The PAID-T has shown psychometric validity with sensitivity to change in symptoms [32].

Diabetes Strengths and Resilience (D-STAR) is a 12-item questionnaire to assess the youth’s confidence in managing their diabetes. The instrument has shown adequate reliability as well as construct and criterion validity. Items capturing diabetes-related confidence, getting help with diabetes management, and disclosure/social support are rated on a 5-point Likert scale (1 = never to 5 = almost always). Total scores range from 12 to 60, and higher scores suggest greater strength and resilience [33].

The Diabetes Family Conflict Scale (DFCS) uses 19 items to assess diabetes family conflict. Youth and family members each completed questionnaires regarding how much they argued about items such diabetes management (food, glucose monitoring, taking medications, managing symptoms, appointments) and talking to others about diabetes (friends, relatives, teachers) in the past month [34]. Items are scored on a 3-point Likert scale (1 = never argue to 3 = always argue). Total scores range from 19 (no conflict) to 57 (high level of conflict) [34]. The DFCS has shown acceptable internal reliability for both youth and parent reports [35].

The Diabetes Family Responsibility Questionnaire (DFRQ) uses 17 items to assess how youth with T1D and their families share responsibilities around diabetes management. Youth and family members each completed questionnaires that assess diabetes management (food, glucose monitoring, taking medications, managing symptom appointments) and talking to others about diabetes (friends, relatives, teachers). Items are scored on a 3-point Likert scale (1 = youth taking or initiating responsibility for this task almost all the time; 2 = family and youth sharing responsibility for this task almost equally; 3 = family taking or initiating responsibility for this task almost all of the time). Scores can range from 17 (youth’s responsibility) to 51 (family member’s responsibility), where the midpoint (34) represents equal responsibility [36]. In a prior study of youth and their family, internal consistency was found to be acceptable for both groups (youth *α* = 0.74; family member *α* = 0.77) [37].

The adapted Health Care Climate Questionnaire (HCCQ) uses 6 items to assess the patient’s perception of their healthcare autonomy and support regarding their last visit with their doctor. Items about feeling understood, being provided choices, the confidence to make changes, being encouraged to ask questions, and being listened to are scored on a 7-point Likert scale (1 = strongly disagree to 7 = strongly agree). Higher scores indicate greater perception of support [38]. Similar short versions of the HCCQ used in various populations have demonstrated acceptable to excellent internal consistency (Cronbach’s α 0.72–0.96) and reflected a single-factor structure [38].

### 2.7. Compensation and Other Incentives (Study Swag Box)

Each family was eligible to receive up to USD 370 for their participation in focus groups and completion of surveys. Participants received USD 40 gift cards for baseline surveys, USD 20 for visits 1–3 (USD 60 total), USD 60 for visit 4, and USD 10 for the end-of-study survey. Youth and families who participated in focus groups were given USD 30 gift cards. Acknowledging the challenges in maintaining study engagement among this population and understanding the importance of rapport and trust, the study team distributed a “study swag box” to each participant, consisting of stress balls, water bottles, notebooks, stickers, and personalized cards (birthdays, holidays, thank yous). These were provided throughout the study regardless of the youth and family’s assigned group (control or intervention).

### 2.8. Statistical Analysis

Demographic and clinical data were summarized using descriptive statistics (mean and standard deviation (SD), median and range, or count and percentage). Categorical variables were compared among study groups using Fisher’s exact test. Continuous variables were compared using one-way analysis of variance. Changes in device use were evaluated within participants using McNemar’s Chi-squared tests. Changes in clinical outcomes over the study were compared among study groups using linear mixed models controlling for age and gender, fit via restricted maximum likelihood using a degrees-of-freedom correction appropriate for small samples [39]. *p* values less than 0.05 were considered statistically significant.

## 3. Results

Eighty-two youth and their families were recruited for the study, and seventy-nine completed the study (see CONSORT diagram, Figure S1). The demographic and clinical characteristics of youth who participated in the trial are shown in Table 1, and demographic information from families is shown in Table 2. Youth were similar in terms of age at enrollment, duration of T1D, gender, race or ethnicity, primary language, insurance, diabetes technology use, and HbA1c. Notably, more gender-diverse participants were in standard care (without VPG, 19%) than other study groups, and more racially diverse participants were in standard care with VPG (13% non-Latinx white, compared to 35% overall).

### 3.1. Diabetes Technology Use

At the baseline, most youth reported using CGMs (56, 71%), and fewer reported insulin pump use (36, 46%). Study groups did not significantly differ in terms of device use, although fewer participants in standard care used CGMs (11, 52%) than those in other study groups. At the end of the study, the standard care group included the most frequent adopters of CGMs, with 71% of youth in this group reporting use (15, a 19% increase), on par with other study groups, whose CGM use decreased slightly over the study (McNemar’s χ^2^ = 0.67, *p* = 0.41). Similarly, insulin pump use decreased overall (down to 30 youth, 38%, −8% change), except in the standard care group, where use increased by 5% over the course of the study (overall McNemar’s χ^2^ = 0.89, *p* = 0.35).

Notably, meter use decreased significantly over the course of the study (McNemar’s χ^2^ = 14.00, *p* = 0.0002). Approximately two-thirds of meter users stopped using this form of technology, and most of those (10/17, 59%) adopted newer diabetes technology during the study.

### 3.2. Hemoglobin A1c

**Figure 2:**
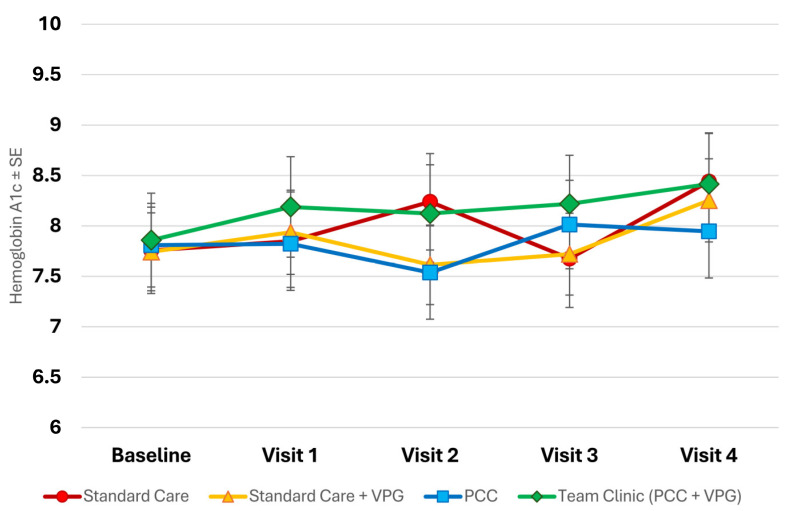
Changes in HbA1c throughout study.

At the baseline, HbA1c levels were similar among all study participants (7.92% on average), and these levels increased for all participants during the study (to 8.58% on average). In unadjusted data, the increase observed in the Team Clinic group (PCC + VPG) was the smallest among the study groups (+0.20%) and the least significant. Although youth in the Team Clinic group entered the study with the highest HbA1c, in both raw and adjusted models, the largest increases in HbA1c were observed among standard care participants (adjusted mean change +0.68%, *p* = 0.05), although this change was not statistically significant (see Figure 2). In adjusted models, all study groups showed non-significant increases in HbA1c over the course of the study.

### 3.3. Depressive Affect

**Figure 3:**
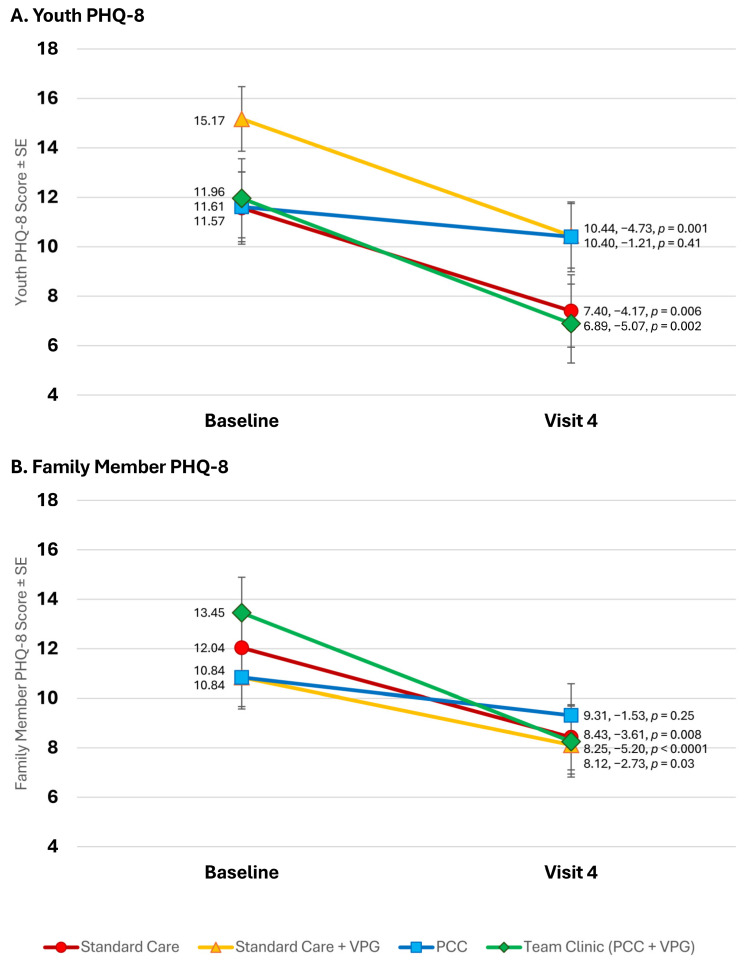
Changes in PHQ-8 depressive affect throughout study.

Nearly all study participants, including both youth and family members, reported significant reductions in depressive affect over the course of the study (see Figure 3A,B). At the baseline, all youth and family reported high rates of depressive affect, consistent with a diagnosis of MDD (≥10). Among youth in standard care with a VPG, PHQ-8 scores (adjusted mean = 15.17) were consistent with a diagnosis of Severe Major Depression. At the end of the study, depressive affect among both family (−3.14, *p* < 0.0001) and youth (−3.76, *p* < 0.0001) was significantly reduced overall. At the end of the study, most participants scored near or below the PHQ-8 cutoff for MDD, including youth in standard care with a VPG (reduced to 10.44, −4.73, *p* = 0.001). Reductions in depressive affect were significant for every study group, except the PCC group, in which it only dropped by −1.21 (*p* = 0.41). In contrast, reductions in depressive affect were the largest among Team Clinic (PCC + VPG) participants, who reported the lowest PHQ-8 scores at the study’s end (reduced to 6.89, −5.07, *p* = 0.002).

### 3.4. Problem Areas in Diabetes (PAID-T)

**Figure 4:**
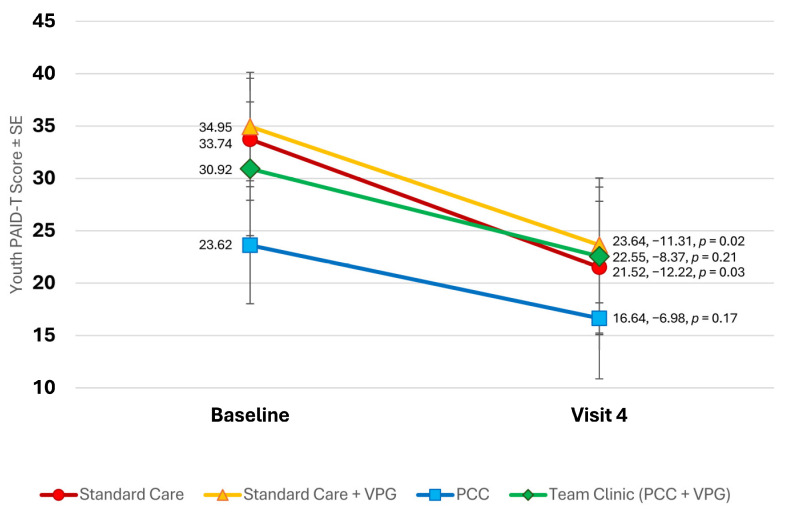
Changes in youth PAID-T scores throughout study.

All youth showed reductions in PAID-T scores, reporting that worries about and complications with diabetes care were less problematic by the end of the study (see Figure 4). Although study groups differed at baseline, they all showed similar changes at the last study visit (−9.86 on average, *p* < 0.0001). Study groups did not differ in terms of changes in PAID-T scores.

### 3.5. T1D-Related Strength and Resilience (D-STAR)

**Figure 5:**
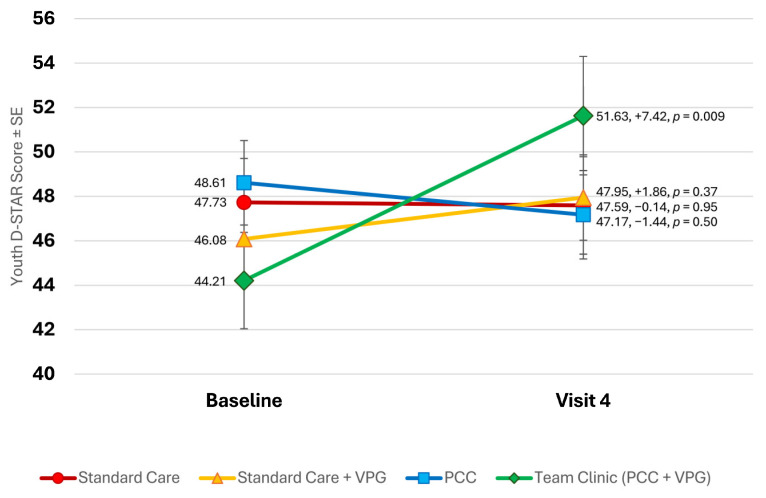
Changes in youth D-STAR scores throughout study.

In contrast, study groups significantly differed in terms of self-perceived strength and resilience concerning T1D care. Participants in both standard care with and without VPG, as well as PCC, reported no significant changes in resilience over the course of the study (see Figure 5). Participants in Team Clinic (PCC + VPG) reported the lowest self-perceived resilience at the baseline (44.21), but also showed the largest gains over the course of the study (+7.42, to 51.63, *p* = 0.009).

### 3.6. Youth- and Family-Perceived Care Responsibility (DFRQ)

**Figure 6:**
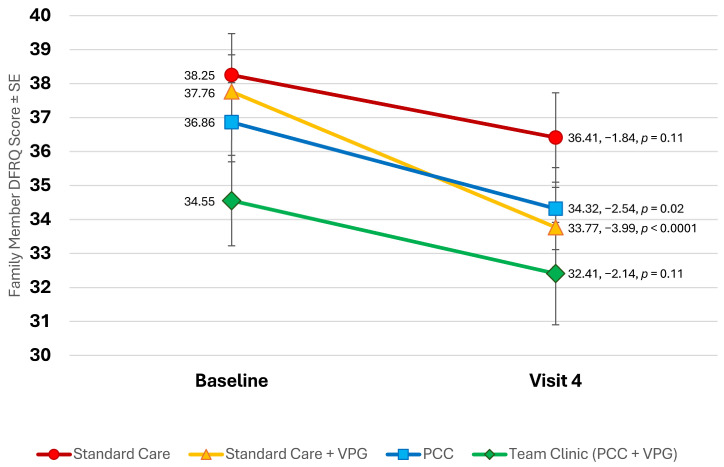
Changes in family DFRQ scores throughout study.

All family members reported significant shifts in care responsibility toward their child over the course of the study (from 37.01 to 34.29 on average, −2.71, *p* < 0.0001; see Figure 6). The largest shifts toward responsibility being attributed to the child were reported by family members of youth in standard care with a VPG (−3.99, *p* < 0.0001), as well as the PCC group (−2.54, *p* = 0.02). Youth in standard care as well as Team Clinic (PCC + VPG) reported similar shifts, although these were not statistically significant. Youth reported smaller, non-significant shifts in care responsibility toward themselves (−0.75, *p* = 0.22) but did not perceive as large of a change as their families perceived.

### 3.7. Other Measures

No significant changes in familial conflict (measured by the DFCS) or healthcare climate (HCCQ) were shown among study participants.

### 3.8. VPG Group Attendance and Appointment Satisfaction

Out of the 39 youth and family members who were invited to attend VPG sessions, 14 youth (36%) and 10 families (26%) did not attend any sessions. Notably, baseline HbA1c values were higher among non-attendees (8.68%) compared to participants who attended at least one youth and/or family session (7.71%; *p* = 0.18), but this difference was not statistically significant. In addition, non-attending families all had private insurance (or both), while all invited participants on public insurance attended VPG sessions. Among youth attendees (n = 25), most attended two sessions (11, 44%), while three (12%) attended all four sessions; among family members (n = 29), an equal number attended all sessions (9, 31%) and no session (9, 31%). The most popular sessions for youth attendees were those discussing proficiency (16, 64%) and energy (15, 60%), followed by balance (9, 36%) and resilience (9, 36%). Family members attended sessions at similar rates (proficiency: 20, 69%; energy: 19, 66%; balance: 17, 59%; resilience: 17, 59%). Both orders of preference reflect the order in which sessions were offered (proficiency first, resilience last).

**Figure 7:**
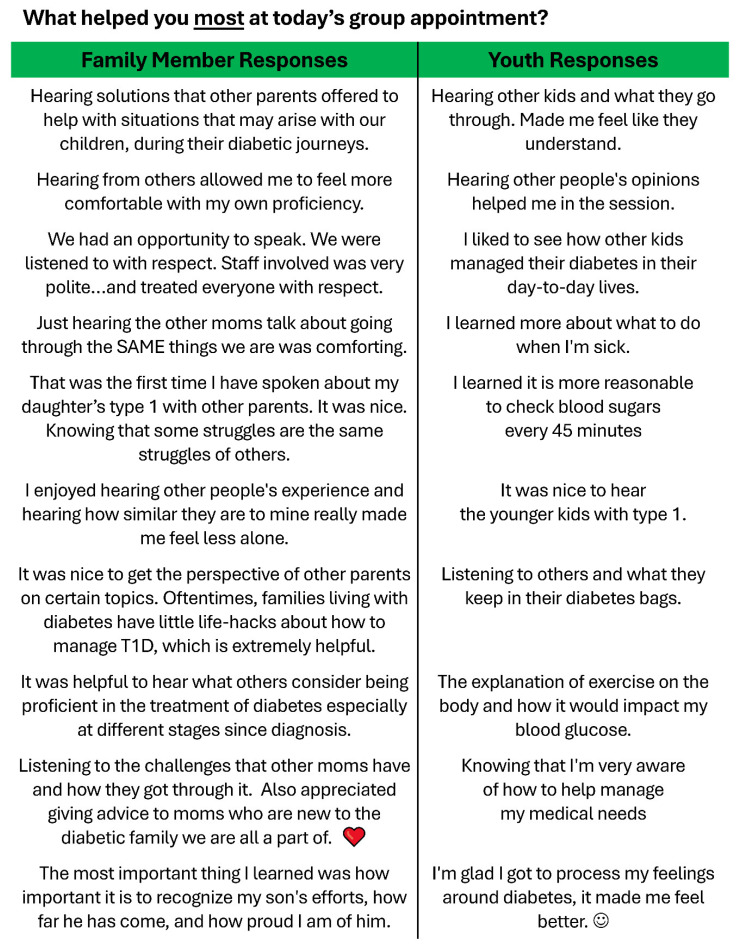
Participant feedback about Team Clinic VPG.

Participants expressed strong satisfaction with the VPG. Youth agreed that they felt comfortable during the VPG, where they learned new information and understood the material covered (average rating 3.52 on a 1–5 scale). In addition, they generally agreed with statements like “I felt supported by other kids in the group visit” and “I would recommend group visits to other kids.” Family members (mean satisfaction = 4.29 on a 1–5 scale) agreed with statements like “I would recommend a group visit to others” and “I liked being with other families during my child’s group visit.” In free text responses, most youth and family members expressed gratitude for the opportunity to talk to others who are in similar situations, bolstered by the reminder that they are not alone in their struggles (Figure 7).

## 4. Discussion

All study participants improved in terms of psychosocial functioning during the study, including reduced depressive affect in both youth and their families, reduced worry about diabetes care issues, and concurrent increases in responsibility for care being placed on the youth, while also showing small increases in HbA1c at the same time. Given that this study occurred during 2022–2023, when the COVID-19 pandemic was waning and society was resuming in-person interactions, the improvements in depressive affect (related to a more active world) and reduced worry about T1D care (as supply chain issues resolved) are understandable. Continuous fluctuations in activities, including eating and exercise, common during the pandemic, may have also contributed to global increases in HbA1c regardless of study group. All youth in the study also showed some greater self-reliance by the end of the study, which could be an age effect or the product of good clinical care and parenting.

Although many effects were observed globally, and likely related to global events, some effects are related to participation in the full intervention, Team Clinic (person-centered care with VPG). In the current study, participants in Team Clinic showed marked increases in resilience as measured by the D-STAR. This is consistent with the clinical orientation of Team Clinic, which stresses the importance of person-centered care and uses empowering language to motivate care adherence and VPG attendance. A supportive group of peers may derive strength through sharing their stories about living with T1D, amplifying feelings of resilience against the condition.

Notably, this effect was not present in the group that received person-centered care alone (without VPG), as though exposure to empowering language from one’s clinician only is not enough to instill change. This deficit may be attributable to the PCC training offered in the present study, which was less structured than what was offered in our previous studies of person-centered VPG care models for adolescents and young adults, which showed positive outcomes for resilience [40,41,42]. In the current study, some clinician trainings (3 out of 11) offered during the height of the COVID-19 pandemic were cancelled. Since COVID-19 was still an acute public health emergency at the time of the study, institutional barriers and overcommitted staff may have contributed to the poor performance of the PCC group.

In some ways, this is consistent with our previous work on young adults, which showed changes in resilience were not associated with participation in a person-centered intervention. However, in pilot studies of that intervention, participants who attended more VPGs reported increased levels of confidence measured by the D-STAR [43]. In the current study, we have demonstrated for a second time an association between increased engagement in peer groups and increased levels of resilience. This theme of resilience may be especially important to individuals with T1D who have been historically marginalized by the healthcare system and researchers.

Although device use dropped slightly over the course of the study while HbA1c increased, these did not occur in the same participants. Standard care participants were the most likely to adopt CGM during the study, but this care group also experienced the largest HbA1c increases over the course of the study. In other words, the use of devices could have been in response to increasing HbA1c levels or a desire to change diabetes care, instead of being used to prevent increases in those levels. Nevertheless, the adoption of diabetes technology is associated with improved long-term outcomes [44], so this improvement in the standard care group is laudable. In addition, many participants stopped using meters, and most replaced the technology with a newer form (CGM or insulin pump).

Many improvements seen in this study impacted all participants, suggesting that there is value in adherence to any evidence-based care model delivered in a structured environment like a clinical trial. In the pragmatic Team Clinic trial, youth were not randomized to new clinicians intentionally. Instead, they were allowed to maintain their relationships with their existing diabetes care clinicians. This study design is based on extensive patient feedback, which stresses the importance of the clinician and family relationship over methodological rigor. Some youth may prefer the support of Team Clinic, while others may prefer the structure of standard care; in either case, those participating in preferred care models are likely to stay engaged and realize better outcomes. The Team Clinic model has been evaluated in a real-world setting by participants who made real-world choices (as opposed to being randomized), and results obtained when this intervention is deployed in other clinics can be expected to resemble what is seen here.

Participants were randomized to participate in the VPG or not, and the benefits and impact on reinforcing resilience are clear. All youth randomized to the VPG were invited, but attendance varied between those that were publicly insured and privately insured. All publicly insured youth attended, which demonstrates the viability of VPGs as an effective care model for individuals impacted by social determinants of health. Those encountering the highest barriers and greatest needs may find connection and support in VPGs, or they might be searching for new care models because standard care models are not serving them. Additionally, young people and families impacted by social determinants of health may not have the luxury of leisure time for social activities and fostering relationships, especially those that support someone managing a chronic disease. Privately insured participants may have had additional resources, (e.g., mental health support, social and online communities, paid time off for self or family) that offset the necessity of participation in a VPG as frequently. Although public insurance was not a barrier to receiving benefits from the VPG in the current study, some participants may still need additional support to overcome barriers to study participation. We must continue to consider the best way to engage communities who have historically been left out of research and innovative clinical care models, as these are the individuals who need it the most.

VPGs may have also mitigated an unintended effect of the person-centered care intervention, as those randomized to receive Team Clinic (PCC + VPG) showed the largest decreases in depressive affect at the end of the COVID-19 pandemic, while those in PCC alone showed much smaller decreases (and no iatrogenic increases). Since changes in depressive affect were also seen in the standard care groups (with or without VPG), this shift is likely a global effect rather than an intervention effect. However, peer groups may have been important venues for youth to discuss issues that came up in the context of person-centered care (e.g., a focus on how interpersonal stressors are impacting T1D care), and their lack of availability to some participants may have been unintentionally detrimental. Nevertheless, the impact of person-centered care is likely synergistic with the effects of peer support groups, whether in person or virtual.

The importance of peer groups is most strongly reflected in the feedback offered by participants (Figure 7). Nearly all youth and family members expressed appreciation for being given the opportunity to be heard and to share their struggles with others. They may not find a perfect answer, although some report this outcome, but all are thankful that they are not alone in their struggles. Marginalized families may feel a sense of unbelonging or loneliness in unfamiliar medical care settings, and Team Clinic addresses those feelings, while also providing practical guidance on T1D care practices.

### Limitations

While our study resulted in positive outcomes, limitations exist. More ethnically and racially diverse individuals participated in this study in comparison to the previous Team Clinic study conducted in Colorado. However, generalizability is most likely impacted by the exclusion of monolingual Spanish speaking families. Most of the patients seen in our clinic are English-speaking and/or bilingual. However, we do serve some monolingual Spanish-speaking families who may have benefitted from this study. Since our hospital is in an urban location and serves unique patients, this model may not be applicable to some patients who live in rural areas. There may also be selection bias, since we only recruited in one location, and those who volunteered to be in the study may differ from those that declined. Our study was also impacted by the COVID-19 pandemic, which may have affected clinical outcomes like HbA1c. Further, the PCC groups (PCC and Team Clinic) had slightly fewer participants. Although our hospital serves primarily publicly insured patients, more families with private insurance were recruited successfully, which may have impacted our results or their generalizability.

## 5. Conclusions

Team Clinic is an alternative care model that demonstrated effectiveness in addressing the care needs of racially, ethnically, and economically diverse youth with T1D. Although most clinical benefits were seen among all study participants, Team Clinic bestowed unique benefits on its participants, including increased self-perceived strength and resilience in the face of challenges related to T1D. Participation in virtual peer groups demonstrated benefits, but it also provided a much-needed space for connection, comfort, support, and—most importantly for youth and families living with T1D—to realize that they are not alone. As dedicated clinicians caring for young people and families living with T1D, we must continue to redesign our models and listen to the needs in our community.

## 6. Future Directions

The research team will continue reviewing data and use the feedback gathered from all stakeholders to further adapt Team Clinic. With access to our in-person and virtual Team Clinic toolkits, future iterations may also be examined in different clinical care settings across the nation. Additionally, we hope to reassess our in-person Team Clinic model and consider options for hybrid and modifiable implementation (e.g., in-person model to start, VPG on a drop-in/as-needed basis, in-person model for major transitions in care, etc.). Models with more evening and weekend choices will be developed to support the busy lives of youth and families, especially those who do not have the luxury of paid time off from work or routine childcare. Our routine clinical care model and standard hours are not conducive to collaborative and inclusive care. Young people and families living with diabetes need choice and empowerment in their diabetes care, including the methods, times, and models in which they receive care. The team hopes to build on our current experiences and data; continue partnering with youth and families living with diabetes, especially those from marginalized and historically excluded communities; and conduct additional research with a focus on patient and family choice and attention to what they tell us they need and want from their diabetes care. Finally, we want to consider populations within our diabetes community who might benefit from an adapted Team Clinic model, such as young people living with autism and T1D, families managing complex mental or physical conditions and T1D, refugee families with T1D moving into a new community, or families who otherwise need T1D care but do not speak English.

## Figures and Tables

**Table 1:** Youth demographic and clinical characteristics.

Characteristic Mean (SD) or n (Column %)	Total	Study Group				*p* Value
		Standard Care	Standard Care + VPG	PCC	Team Clinic (PCC + VPG)	
Youth	79	21	23	19	16	
Age at Enrollment	13.34 (1.62)	13.61 (1.29)	13.07 (1.81)	12.89 (1.63)	14.00 (1.56)	0.96
T1D Duration	4.33 (2.78)	3.39 (2.40)	4.25 (3.41)	4.72 (2.56)	5.07 (2.18)	0.33
Gender						
Female	37 (47)	9 (43)	11 (48)	10 (53)	7 (44)	0.70
Male	32 (41)	6 (29)	10 (44)	8 (42)	8 (50)	
Non-Binary/Other	7 (9)	4 (19)	1 (4)	1 (5)	1 (6)	
Unknown	3 (4)	2 (11)	1 (4)	0	0	
Race and Ethnicity						
Asian	6 (8)	0	3 (13)	2 (11)	1 (6)	0.32
Black/African-Amer.	5 (6)	1 (5)	3 (13)	0	1 (6)	
Hawaiian/Pacific Isl.	1 (1)	0	1 (4)	0	0	
Latinx/Hispanic	27 (34)	8 (38)	8 (35)	7 (37)	4 (25)	
Non-Latinx white	28 (35)	8 (38)	3 (13)	9 (47)	8 (50)	
Multiracial	4 (5)	1 (5)	3 (13)	0	0	
Other/Unknown	8 (10)	3 (14)	2 (9)	1 (5)	2 (13)	
Primary Language						
English	73 (92)	18 (86)	22 (96)	18 (95)	15 (94)	0.66
Spanish	3 (4)	1 (5)	0	1 (5)	1 (6)	
Other/Unknown	3 (4)	2 (10)	1 (4)	0	0	
Bilingual	22 (28)	7 (33)	7 (30)	4 (21)	4 (25)	0.91
Insurance						
Public (Medi-Cal, CCS)	16 (20)	6 (29)	3 (13)	4 (21)	3 (19)	0.29
Private (HMO, PPO)	37 (47)	9 (43)	8 (35)	12 (63)	8 (50)	
Both	26 (33)	6 (29)	12 (52)	3 (16)	5 (31)	
A1c						
Baseline	7.92 (1.88)	8.00 (1.86)	7.80 (1.48)	7.81 (2.17)	8.08 (2.18)	0.96
Study End	8.58 (1.92)	8.82 (2.17)	8.45 (2.18)	8.76 (2.05)	8.28 (0.97)	0.91
CGM Use						
Baseline	56 (71)	11 (52)	17 (74)	16 (84)	12 (75)	0.32
Study End	52 (66)	15 (71)	14 (61)	13 (68)	10 (63)	0.94
Insulin Pump Use						
Baseline	36 (46)	7 (33)	9 (39)	9 (47)	11 (69)	0.31
Study End	30 (38)	8 (38)	6 (26)	8 (42)	8 (50)	0.68
Meter Use						
Baseline	25 (32)	9 (43)	8 (35)	5 (26)	3 (19)	0.51
Study End	8 (10)	4 (19)	1 (4)	3 (16)	0	0.33

**Table 2:** Family demographic characteristics.

Characteristic Mean (SD) or n (Column %)	Total	Study Group				*p* Value
		Standard Care	Standard Care + VPG	PCC	Team Clinic (PCC + VPG)	
Family Member	79	21	23	19	16	
Race and Ethnicity						
Asian	5 (6)	0	2 (9)	2 (11)	1 (6)	0.97
Black/African-Amer.	5 (6)	2 (10)	2 (9)	0	1 (6)	
Latinx/Hispanic	30 (38)	8 (38)	8 (35)	8 (42)	6 (38)	
Non-Latinx white	32 (41)	10 (48)	8 (35)	7 (37)	7 (44)	
Multiracial	1 (1)	0	1 (4)	0	0	
Other/Unknown	6 (8)	1 (5)	2 (9)	2 (11)	1 (6)	
Primary Language						
English	68 (86)	15 (71)	21 (91)	18 (95)	14 (88)	0.09
Spanish	6 (8)	4 (19)	0	0	2 (13)	
Other/Unknown	5 (6)	2 (10)	2 (9)	1 (5)	0	
Bilingual	34 (43)	10 (48)	7 (30)	10 (53)	7 (44)	0.70
Parental Education (Highest)						
Some High School	5 (6)	3 (14)	0	0	2 (13)	0.57
High School Diploma	8 (10)	4 (19)	1 (4)	1 (5)	2 (13)	
Trade/Technical	8 (10)	2 (10)	4 (17)	1 (5)	1 (6)	
Some College	18 (23)	1 (5)	8 (35)	5 (26)	4 (25)	
Bachelor’s Degree	17 (22)	5 (24)	4 (17)	5 (26)	3 (19)	
Graduate Degree	16 (20)	4 (19)	4 (17)	5 (26)	3 (19)	
Other/Unknown	7 (9)	2 (10)	2 (9)	2 (11)	1 (6)	
Marital Status						
Single	13 (16)	6 (29)	1 (4)	2 (11)	4 (25)	0.45
Married/Civil Union	44 (56)	8 (38)	15 (65)	11 (58)	10 (63)	
Divorced	11 (14)	3 (14)	3 (13)	3 (16)	2 (13)	
Widowed	3 (4)	0	1 (4)	2 (11)	0	
Separated	3 (4)	1 (5)	1 (4)	1 (5)	0	
Living with Partner	3 (4)	2 (10)	1 (4)	0	0	
Unknown	2 (3)	1 (5)	1 (4)	0	0	

## Data Availability

Study data are not publicly available due to HIPAA, Data User Agreements, and the consent process. Data are only shared with the research team and funder.

## Supplementary Materials

The following supporting information can be downloaded at: https://www.mdpi.com/article/10.3390/children11111383/s1, Figure S1. CONSORT Diagram.
